# The Combination of AHCC and ETAS Decreases Migration of Colorectal Cancer Cells, and Reduces the Expression of *LGR5* and *Notch1* Genes in Cancer Stem Cells: A Novel Potential Approach for Integrative Medicine

**DOI:** 10.3390/ph14121325

**Published:** 2021-12-18

**Authors:** Francesca Paganelli, Francesca Chiarini, Annalisa Palmieri, Marcella Martinelli, Paola Sena, Jessika Bertacchini, Luca Roncucci, Alessandra Cappellini, Alberto M. Martelli, Massimo Bonucci, Carla Fiorentini, Ivano Hammarberg Ferri

**Affiliations:** 1 Department of Biomedical and Neuromotor Sciences, University of Bologna, 40126 Bologna, Italy; francesca.paganell16@unibo.it (F.P.); alessandra.cappellini@unibo.it (A.C.); alberto.martelli@unibo.it (A.M.M.); 2 CNR-Institute of Molecular Genetics “Luigi Luca Cavalli-Sforza”, Unit of Bologna, 40136 Bologna, Italy; 3 IRCCS Istituto Ortopedico Rizzoli, 40136 Bologna, Italy; 4 Department of Experimental, Diagnostic and Specialty Medicine, University of Bologna, 40138 Bologna, Italy; annalisa.palmieri@unibo.it (A.P.); marcella.martinelli@unibo.it (M.M.); 5 Department of Surgery, Medicine, Dentistry and Morphological Sciences with Interest in Transplant, Oncology and Regenerative Medicine, University of Modena and Reggio Emilia, 41125 Modena, Italy; paola.sena@unimore.it (P.S.); jessika.bertacchini@unimore.it (J.B.); 6 Department of Diagnostic, Clinical and Public Health Medicine, University of Modena and Reggio Emilia, 41124 Modena, Italy; luca.roncucci@unimore.it; 7 Association for Research on Integrative Oncology Therapies (ARTOI), 00165 Rome, Italy; maxbonucci@artoi.it (M.B.); dottivanoferri@gmail.com (I.H.F.)

**Keywords:** natural supplements, combined treatments, cancer cell migration, cancer stem cells

## Abstract

The AHCC standardized extract of cultured *Lentinula edodes* mycelia, and the standardized extract of *Asparagus officinalis* stem, trademarked as ETAS, are well known supplements with immunomodulatory and anticancer potential. Several reports have described their therapeutic effects, including antioxidant and anticancer activity and improvement of immune response. In this study we aimed at investigating the effects of a combination of AHCC and ETAS on colorectal cancer cells and biopsies from healthy donors to assess the possible use in patients with colorectal cancer. Our results showed that the combination of AHCC and ETAS was synergistic in inducing a significant decrease in cancer cell growth, compared with single agents. Moreover, the combined treatment induced a significant increase in apoptosis, sparing colonocytes from healthy donors, and was able to induce a strong reduction in migration potential, accompanied by a significant modulation of proteins involved in invasiveness. Finally, combined treatment was able to significantly downregulate *LGR5* and *Notch1* in SW620 cancer stem cell (CSC) colonospheres. Overall, these findings support the potential therapeutic benefits of the AHCC and ETAS combinatorial treatment for patients with colorectal cancer.

## 1. Introduction

Colorectal cancer (CRC) is the fourth most common neoplasia throughout the world [1]. Localized CRC is often successfully treated with curative surgery followed by chemotherapy in patients at high risk for recurrence [2]. However, because early-stage CRC can be asymptomatic, a minority (40%) of CRC cases are diagnosed early, resulting in a large number of patients initially diagnosed with metastatic CRC, for whom the 5-year survival rate is very poor (13%) [3]. Chemotherapy is the standard treatment for CRC patients, but its systemic toxicity encourages the use of integrative medicine to alleviate symptoms correlated to cytotoxicity and to possibly potentiate chemotherapy efficacy.

Integrative medicine (IM) is based on the use of alternative therapies that have high-quality scientific evidence of safety and efficacy, in combination with conventional medical therapies [4]. Due to the constant demand of new therapies for cancer patients, benefits of IM were widely evaluated in cancer treatments [5]. In recent years, naturally-derived compounds and their properties have been considered as potential anticancer and immune-stimulatory adjuvants. Medicinal plants traditionally are a source of active compounds that could be employed as supplements for conventional chemotherapies [6,7]. Among these compounds, AHCC^®^, a standardized extract of cultured *Lentinula edodes* mycelia, is currently being assessed for its innate properties as a potential adjuvant to conventional therapies [8]. AHCC is composed of various small molecular weight oligosaccharides, polysaccharides, amino acids, lipids, and minerals [8,9,10]. The principal component is α-glucan, well known for its immunomodulatory effects [8,9,10]. For this reason, AHCC has been widely used as supplement in immunocompromised people, including cancer patients, and several studies reported the beneficial effects of AHCC in cancer treatment. AHCC delayed melanoma development in mice models regulating both innate and adaptive immune responses [11]. Moreover, this compound was able to inhibit the proliferation of ovarian cancer cells and human colon adeno-carcinoma cells and inhibited intestinal tumor progression in mice [12,13]. AHCC also had beneficial effects in pancreatic cancer cells, downregulating the heat shock factor 1, responsible for therapy resistance in this tumor [14]. AHCC use also showed efficacy in reducing chemotherapy-induced adverse effects in cancer patients, alleviating bone marrow suppression, hepatotoxicity and nephrotoxicity [15,16,17,18]. Encouraging effects of AHCC have also been demonstrated in chronic diseases [19] and in host protection during bacterial [20] and viral infections [21]. In the context of natural compounds as supportive care for several diseases, there are also emerging evidences about ETAS^®^, a standardized extract of *Asparagus officinalis* stem. The extract contains 3-Alkyldiketopiperazines (Asparaprolines), which are involved in the regulation of heat shock protein (HSP)70 expression [22]. Moreover, ETAS downregulated HSP27 in pancreatic tumor cells, which is normally linked to gemcitabine resistance in this type of cancer [23]. Previous studies demonstrated that ETAS also exerted neuroprotective effects, regulating neurological anti-ageing responses and normalizing circadian rhythm signaling [24,25]. Additionally, ETAS administration prevented pro-inflammatory responses induced by reactive species of oxygen [26,27]. 

Here we evaluated the effects of a combination of AHCC and ETAS on colon cancer cell lines and biopsies from healthy donors. We found that the combination of AHCC and ETAS was able to reduce cell viability of colon cancer cells, while sparing colonocytes from biopsies of healthy donors. Moreover, the reduction in cell viability was accompanied by a significant induction of apoptosis in colon cancer cell lines, with activation of caspases. The combined treatment was also able to reduce migration abilities, inducing a significant decrease in ROCK2 (Rho Associated Coiled-Coil Containing Protein Kinase 2) and metalloproteinase (MMP9), and a significant increase in E-cadherin protein expression. Finally, in cancer stem cells (CSCs) of tumorspheres from SW620, the combined treatment of AHCC and ETAS caused a significant reduction in the gene expression of *LGR5* and *Notch1*, opening new perspectives for the testing of these compounds in association with conventional chemotherapy.

## 2. Results

### 2.1. The Combination of AHCC and ETAS Is Synergistic and Inhibits Cell Growth in Colon Cancer Cells 

**Figure 1:**
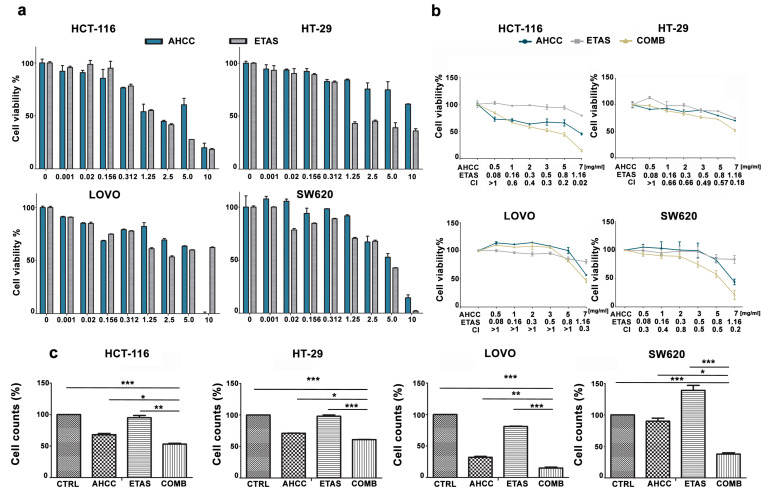
AHCC and ETAS combined treatment inhibits CRC cells growth. (**a**) MTT analyses were performed to assess cell viability of colon cancer cell lines treated for 48 h with increasing concentrations of AHCC and ETAS as single agents. Results are the mean of at least three independent experiments ± SD; (**b**) MTT analyses were performed to assess cell viability of colon cancer cell lines treated for 48 h with AHCC and ETAS as single agents or in combination at a fixed ratio (ratio 6:1). Concentrations of compounds used in each single point are reported in the graph. A combination index (CI) was calculated with CompuSyn software and values were plotted as shown in the graph; CI < 0.9 indicates synergism. Results are the mean of at least three independent experiments ± SD; (**c**) Flow cytometry absolute counts of viable colon cancer cells treated for 48 h with AHCC and ETAS as single agents or in combination (COMB). For HCT-116, HT-29, and SW620 cells, we used 3 mg/mL AHCC + 0.5 mg/mL ETAS. For LOVO cells we employed 7 mg/mL AHCC + 1.16 mg/mL ETAS. “CTRL” indicates non-treated cells used as control cells. The mean ± SD of three independent experiments is plotted. Asterisks indicate statistically significant differences between samples: * *p* < 0.05, ** *p* < 0.01, *** *p* < 0.001.

To assess the effects of AHCC and ETAS, we incubated colon cancer cells for 48 h with increasing concentrations of these compounds as single agents. We then analyzed the rate of cell viability through MTT assays (Figure 1a). AHCC induced a significant reduction of 50% of cell viability when used at the concentrations of 2.5–5 mg/mL in HCT-116 cells but higher AHCC concentrations were required to significantly affect cell viability in LOVO, HT-29, and SW620 cells, after 48 h of treatment (Figure 1a).

ETAS treatment was able to significantly reduce the viable cell number in all cell lines tested. In particular, HT-29 cells were the most sensitive, with an IC_50_ lower than 1.25 mg/mL, while significant effects on viability were evident between 2.5 and 5 mg/mL in HCT-116, LOVO and SW620 cells (Figure 1a). LOVO cells did not show further effects employing increasing concentrations of ETAS (Figure 1a). 

We also evaluated the effects of AHCC and ETAS in combination at a fixed ratio (6:1). MTT analysis demonstrated that the combined treatment was synergistic in inducing cytotoxicity, affecting cell viability in all cell lines tested (Figure 1b). The combination index (CI) values, calculated with CompuSyn software for dose-effect analysis, indicated the existence of a synergism (CI < 0.9) in SW620 cells in all combinations tested, while HCT-116 and HT-29 cells showed synergistic effects in all combinations tested except for the lowest one (Figure 1b). We also found a synergistic effect of the combination of AHCC and ETAS in LOVO cells at 7 mg/mL AHCC and 1.16 mg/mL ETAS (Figure 1b). Then, cell counts were performed after treating colon cancer cells with AHCC or ETAS as single agents and in combination (at a ratio of 6:1) for 48 h. We employed AHCC and ETAS at 3 mg/mL AHCC + 0.5 mg/mL ETAS for HCT-116, HT-29 and SW620, and at 7 mg/mL AHCC + 1.16 mg/mL ETAS for LOVO cells. The combination of AHCC and ETAS employed for each cell line was chosen on the basis of the previous synergistic effects obtained treating cells with AHCC and ETAS combined at a fixed ratio.

This combination was able to induce a significant decrease in cell proliferation in all cell lines analyzed, demonstrating that the combination was effective in inducing an arrest in cancer cell growth (Figure 1c).

### 2.2. The Combination of AHCC and ETAS Does Not Affect Viability of Human Colonocytes from Healthy Donors 

**Figure 2:**
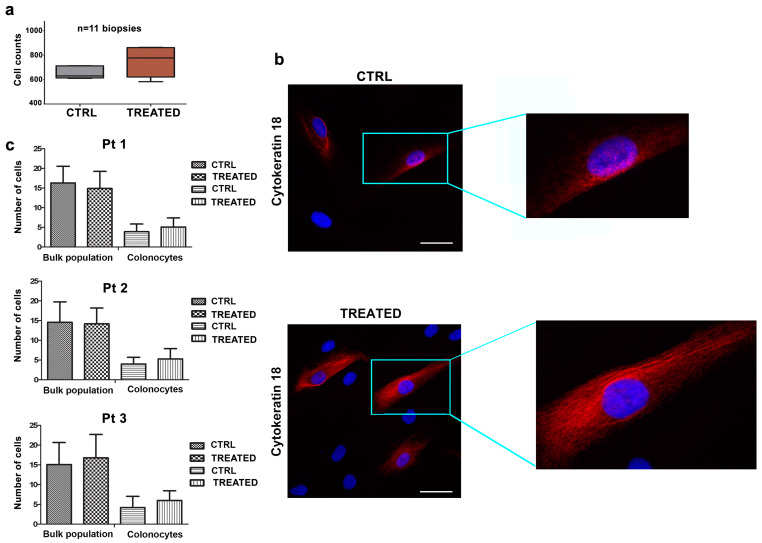
The combined treatment of AHCC and ETAS has no effects on healthy human colonocytes. (**a**) Cell viability assays in human primary cell cultures treated with a combination of AHCC and ETAS for 48 h (3 mg/mL for AHCC and 0.5 mg/mL for ETAS). Results are presented as the mean of three independent experiments ± SD conducted on normal mucosal biopsies of eleven patients; (**b**) Immunofluorescence analysis of Cytokeratin-18 filament bundles well recognizable within the cytoplasm of colonocytes in untreated samples and treated with the combined AHCC and ETAS samples. Magnification 40×, scale bar 10 μm; (**c**) Cell counts of cells in biopsies from three patients seeded on glass slides and treated for 48 h with a combination of 3 mg/mL for AHCC and 0.5 mg/mL ETAS. The mean ± SD of 3 patients is shown.

The effects of combined treatment with AHCC and ETAS were evaluated in primary cell cultures, obtained from biopsies of colorectal normal mucosa, using cell viability assays. To this aim, specimens from eleven healthy patients were employed. Cells from biopsies were treated with AHCC and ETAS for 48 h. As shown in Figure 2a, the effects of the combined treatment did not significantly influence cell viability.

Due to the fact that the biopsies were enriched in fibroblasts, stromal cells, and colonocytes, we performed immunofluorescence experiments to evaluate the effects of the combination of AHCC and ETAS on the bulk population and on colonocytes. Samples from 3 healthy donors were cultured on coverslip glasses and treated for 48 h, and colonocytes were then marked with Cytokeratin-18 antibody, a specific marker for epithelial cells (Figure 2b). Our results indicated that there was no statistically significant difference in cell number in treated bulk population versus untreated cells (Figure 2c). Moreover, the number of colonocytes treated with the combination of AHCC and ETAS was unaffected by the treatment, indicating that the combination of AHCC and ETAS was not able to induce modulation in cell viability or cell number in cells from biopsies (Figure 2c).

### 2.3. The Combination of AHCC and ETAS Promotes a Significant Increase in Cell Apoptosis in Colon Cancer Cells

**Figure 3:**
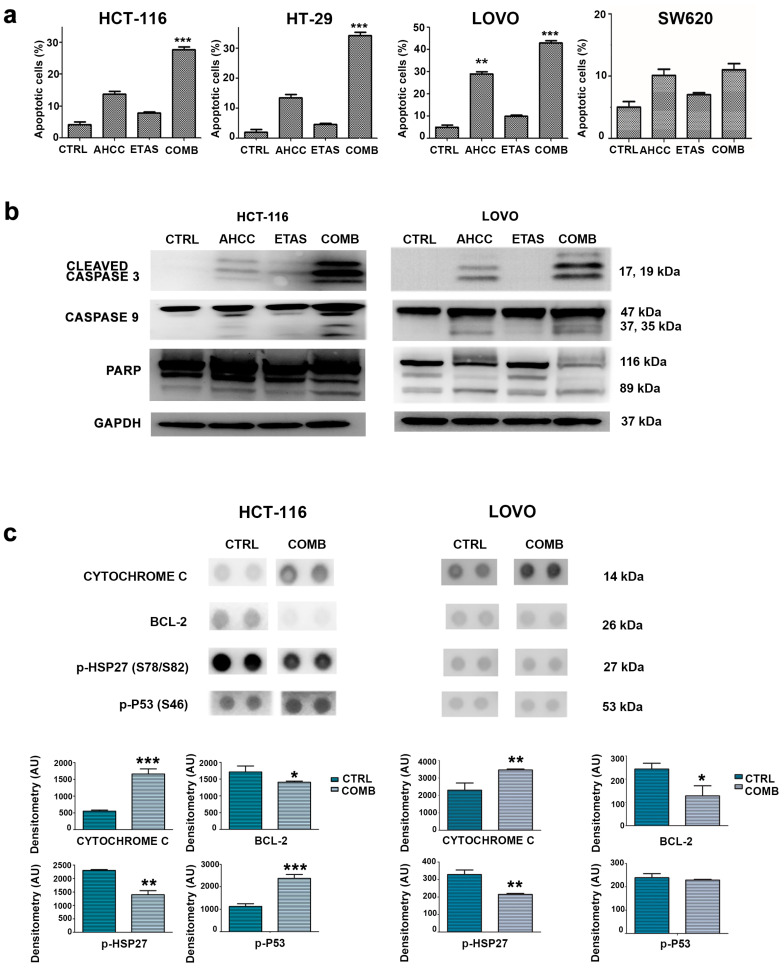
AHCC and ETAS combined treatment induces apoptosis in CRC cells. (**a**) Apoptosis analysis of colon cancer cell lines treated for 48 h with AHCC and ETAS as single agents or in combination (COMB). For HCT-116 and SW620 cells, we used 3 mg/mL AHCC + 0.5 mg/mL ETAS. For HT-29 and LOVO cells we employed 7 mg/mL AHCC + 1.16 mg/mL ETAS. CTRL indicates untreated cells. The percentage of apoptotic cells is plotted as the mean of three independent experiments ± SD. Asterisks indicate statistically significant differences with respect to untreated cells, ** *p* < 0.01, *** *p* < 0.001; (**b**) Western blotting analysis of cleaved caspase 3, cleaved caspase 9 and PARP protein expression in HCT-116 and LOVO cell lines, treated with AHCC and ETAS as single agents or in combination (COMB). For HCT-116 we used 3 mg/mL AHCC + 0.5 mg/mL ETAS; for LOVO we used 7 mg/mL AHCC + 1.16 mg/mL ETAS. GAPDH was used as the loading control; (**c**) Stress-protein array analysis in HCT-116 and LOVO cells. Each treated sample (COMB; for HCT-116 we used 3 mg/mL AHCC + 0.5 mg/mL ETAS; for LOVO we used 7 mg/mL AHCC + 1.16 mg/mL ETAS) is compared to untreated sample (CTRL). Each protein has a double spot. Histograms indicate densitometric analysis. The following proteins were evaluated: Cytochrome C, BCL-2, p-HSP27 (S78/S82) and p-P53 (S46). Asterisks indicate statistically significant differences with respect to control, * *p* < 0.05, ** *p* < 0.01, *** *p* < 0.001.

To evaluate if the effects of AHCC and ETAS on cell viability could be related to apoptosis, flow cytometric analysis was performed. We detected a significant increase in the percentage of apoptotic cells in response to combined treatment in HCT-116, HT-29 and LOVO cell lines (Figure 3a). We also confirmed the enhanced effect of the combined treatment compared to the single ones in inducing apoptosis (Figure 3a). Apoptosis was further investigated by Western blotting analysis, which showed a cleavage of caspase 9, caspase 3, and poly (ADP-ribose) polymerase (PARP) in response to treatment in HCT-116 and LOVO cells (Figure 3b). The most effective modulation was obtained when cells were treated with both AHCC and ETAS (Figure 3b).

To assess which pathway could be involved in AHCC- and ETAS-mediated effects, we performed stress-protein array analyses on HCT-116 and LOVO cell lines treated with AHCC and ETAS in combination for 48 h. We confirmed an induction of apoptosis signaling, as demonstrated by the decrease of BCL-2 protein levels and the increase of Cytochrome C and p(Ser46)p53 protein levels (Figure 3c), well known markers of the apoptotic process [28,29,30]. Interestingly, we also found a significant decrease in HSP27 phosphorylation (Figure 3c). This phosphorylation is linked to HSP27 activation and to apoptosis inhibition. Indeed, the stress condition can activate p38MAPK signaling, which leads to HSP27 phosphorylation and its activation [31]. Activated HSP27 can inhibit apoptosis through inhibition of caspase-9 and caspase-3 cleavage by directly interacting with procaspase-9 and procaspase-3 [32]. Moreover, HSP27 is related to anti-cancer drug resistance and poor prognosis in many types of cancer, including CRC [33,34,35]. Previous studies have also demonstrated that AHCC and ETAS were able to downregulate HSP27 levels in pancreatic cancer cells, enhancing the sensitivity to chemotherapy [23,36]. HSP27 modulation obtained in our model, by treating the cells with the combination of AHCC and ETAS, outlines the potential therapeutic benefits that combined treatment could have in colon cancer patients.

### 2.4. CSC Tumorspheres from CRC Cell Lines Are Affected by AHCC and ETAS Combination

**Figure 4:**
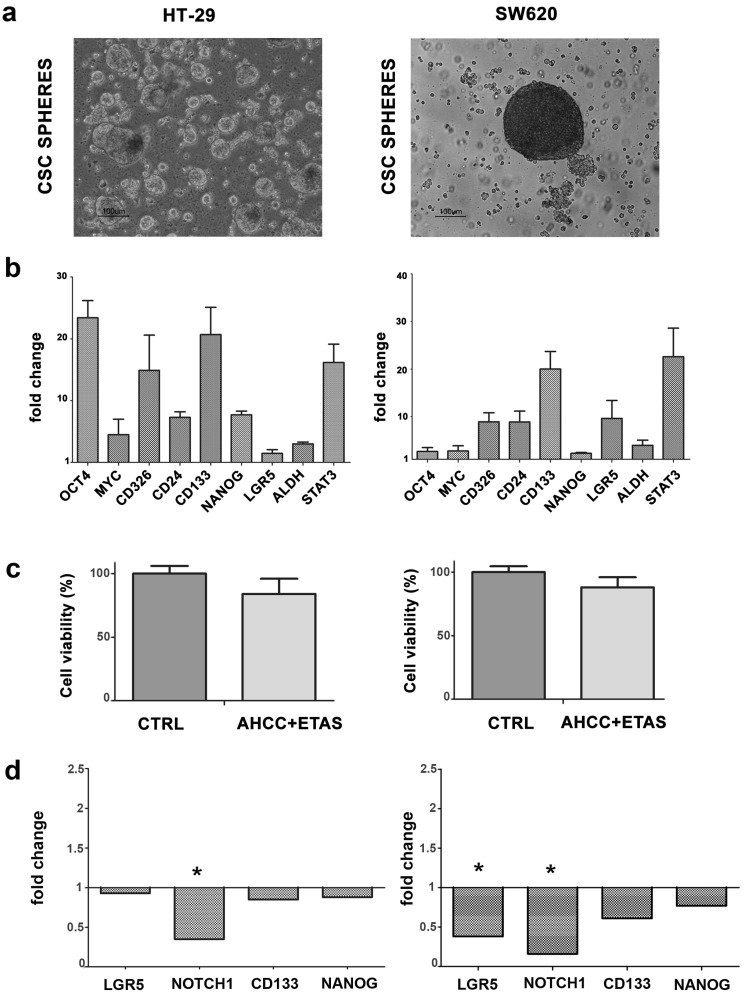
AHCC and ETAS combined treatment affects CSC tumorspheres. (**a**) Representative images show 3D tumorspheres (CSC spheres) suspension cultures derived from HT-29 and SW620 cell lines. Magnification 10×; (**b**) Quantitative RT-PCR analysis of stemness-associated genes (*NANOG*, *OCT4*, *MYC*, *CD326*, *LGR5*, *CD24*, *CD133*, *ALDH* and *STAT3*) in CSC spheres from HT-29 and SW620 compared to 2D parental cell cultures. Fold change is plotted, *p* < 0.05; (**c**) Cell viability in untreated or treated with a combination of AHCC and ETAS (7 mg/mL AHCC + 1.16 mg/mL ETAS) CSC spheres; (**d**) Quantitative RT-PCR analysis of *LGR5*, *Notch1*, *CD133*, and *NANOG* genes in untreated or treated with the combination of AHCC and ETAS CSC spheres. Fold change is plotted, * *p* < 0.05.

Three-dimensional (3D) sphere models are commonly employed to selectively promote the growth of tumor cell populations with stem-like properties, being a functional procedure for the in vitro discovery of new signaling pathways that can drive self-renewal and differentiation in CSCs. In the present work, we employed two established human colon cancer cell lines, HT-29 and SW620 to produce tumorsphere cultures. To this aim, cells were cultivated in non-adherent settings, using serum-free medium supplemented with growth factors. Thus, only cells with stem cell features are likely to proliferate, producing free-floating multicellular spheres. After 1/3 weeks, HT-29, and SW620 cells were able to efficiently form tumorspheres, in agreement with other observations [37]. Cells from spheres were then positively selected with CD326 beads, and allowed to grow as CSC spheres (Figure 4a). To assess the expression profile of the stemness-associated transcripts, we evaluated *NANOG*, *OCT4*, *MYC*, *CD326*, *LGR5*, *CD24*, *CD133*, *ALDH*, and *STAT3* gene expression, compared to 2D parental cell cultures. The expression profile of these transcripts was significantly enriched (*p* < 0.05), further confirming that sphere-forming populations were enriched in CSCs (Figure 4b). 

Then, to determine whether the AHCC and ETAS combination could affect CRC stem-like cells, we treated CSC spheres with the combination of these compounds and we assessed the effects on cell viability. We observed a slight decrease in CSC viability in treated CSC spheres compared to untreated samples (Figure 4c). Finally, we analyzed the expression profile of the stemness-associated transcript *LGR5* (leucine-rich repeat-containing G-protein-coupled receptor 5) and *Notch1* (neurogenic locus notch homolog protein), which are involved in the maintaining of stem cell like properties, and we found a significant decrease in the expression of these genes in SW620 cells, with the same tendency in HT-29 cells (Figure 4d). The treatment was also able to induce a slight downmodulation of *CD133* and *NANOG* in both HT-29 and SW620 cells (Figure 4d). The expression profile of genes involved in cell motility (*ROCK2*, *CDH1*, *SNAI2*, *ITGA5*, *TIMP1*) was also analyzed, finding, however, no significant differences between treated versus untreated CSC spheres (data not shown).

### 2.5. The Combination of AHCC and ETAS Reduces Cell Motility and Migration in Colon Cancer Cells

**Figure 5:**
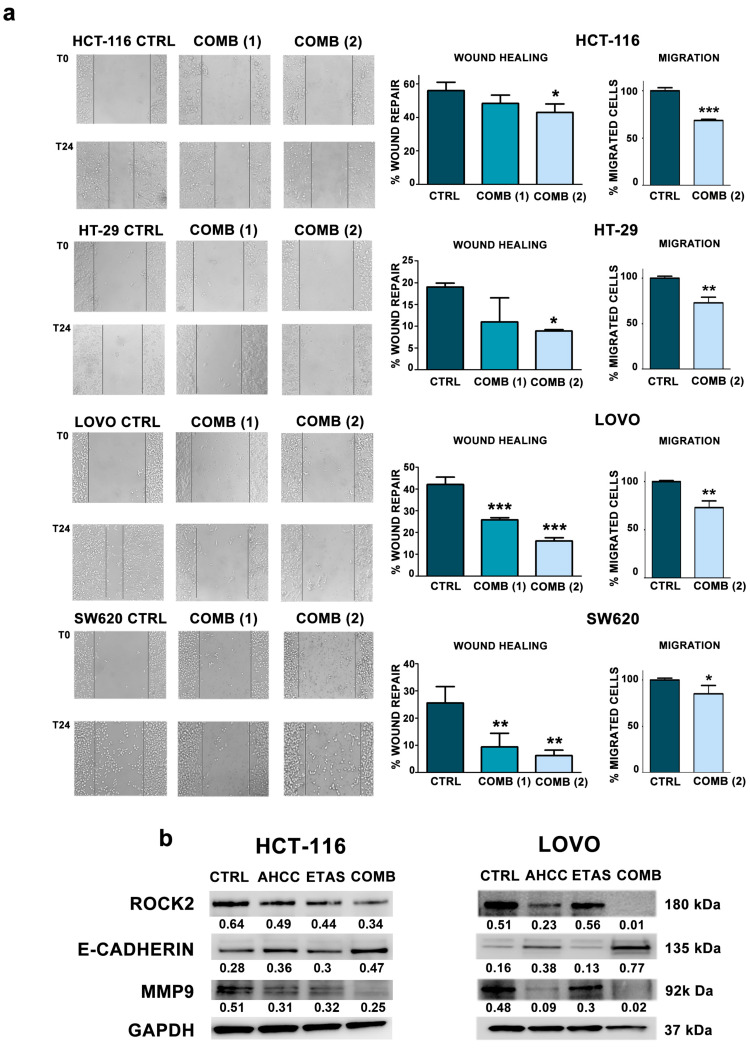
AHCC and ETAS combination significantly reduces CRC cell migration. (**a**) Wound healing and transwell migration assays performed in HCT-116, HT-29, LOVO and SW620 cells treated with increasing concentration of AHCC and ETAS combined treatment (COMB). For combination 1 (COMB (1)), we used 3 mg/mL AHCC + 0.5 mg/mL ETAS. For combination 2 (COMB (2)), we employed 7 mg/mL AHCC + 1.16 mg/mL ETAS. Representative pictures were taken at 0 and 24 h after scratching. For transwell assays, migration was assessed after 24 h of treatment. Magnification 10×. Histograms are plotted as mean ± SD of three independent experiments. Asterisks indicate statistically significant differences with respect to control, * *p* < 0.05, ** *p* < 0.01, *** *p* < 0.001; (**b**) Western blotting analysis of ROCK2, E-cadherin and MMP9 protein expression in HCT-116 and LOVO cell lines, treated with AHCC and ETAS as single agents or in combination (HCT-116 COMB: 3 mg/mL AHCC + 0.5 mg/mL ETAS; LOVO COMB: 7 mg/mL AHCC + 1.16 mg/mL ETAS). GAPDH was used as the loading control. Numbers indicate densitometric analysis plotted as the ratio respect to GAPDH levels.

To understand the role of the combined treatment on cell migration in colorectal cancer cells grown in adherence, we treated the cells with AHCC and ETAS in combination for 24 h and then we assessed migration abilities by wound healing assays. Motility assays demonstrated that cells pretreated with a combination of AHCC and ETAS were significantly less able to migrate compared to untreated cells (Figure 5a). We also performed transwell migration assays, treating the cells with AHCC and ETAS in combination for 24 h. Moreover, to demonstrate the specific effect on motility and migration, we assessed if the concentration used for wound healing assays and transwell assays was able to induce cell death in all cell lines. To this aim we performed annexin V/Propidium Iodide staining in control cells and cells treated for 24 h with the combination of AHCC and ETAS (7 mg/mL AHCC and 1.16 mg/mL ETAS). Our results show that the combination employed for motility and migration assays was ineffective in inducing apoptosis after 24 h of treatment (Supplementary Figure S1). However, we found a significant reduction in migration ability after combined treatment in all cell lines evaluated (Figure 5a). 

Being that the prognosis of CRC metastatic patients was very poor [38], we wanted to investigate the expression of proteins involved in cell migration. We analyzed the expression of several proteins such as ROCK2, MMP9 (matrix metalloproteinase 9) and E-cadherin. The combined treatment caused a significant decrease in ROCK2 and MMP9 protein levels, accompanied by a strong increase of E-cadherin protein levels, compared to single treatments (Figure 5b). These results were very interesting due to the role of these proteins in several tumors. Indeed, ROCK2 is overexpressed and associated with increased invasion and metastasis in breast cancer and bladder cancer [39,40,41]. Moreover, ROCK2 was found to mediate invasion of colon cancer cells [39,40,41]. 

MMP9 downmodulation obtained after the combined treatment is of potential interest because MMP9 is expressed at high levels in CRC patients compared to healthy donors and this overexpression is associated to a worse outcome in these patients [42,43]. 

We also found a strong increase of E-cadherin in cell lines treated with AHCC and ETAS. This protein is an active suppressor of invasion and growth of many epithelial cancers, including colon cancer [44,45]. Indeed, its functional loss represents a key step in the acquisition of the invasive phenotype for several type of tumors [44].

### 2.6. AHCC and ETAS Combination Enhances Oxaliplatinum Effects in Colon Cancer Cells

**Figure 6:**
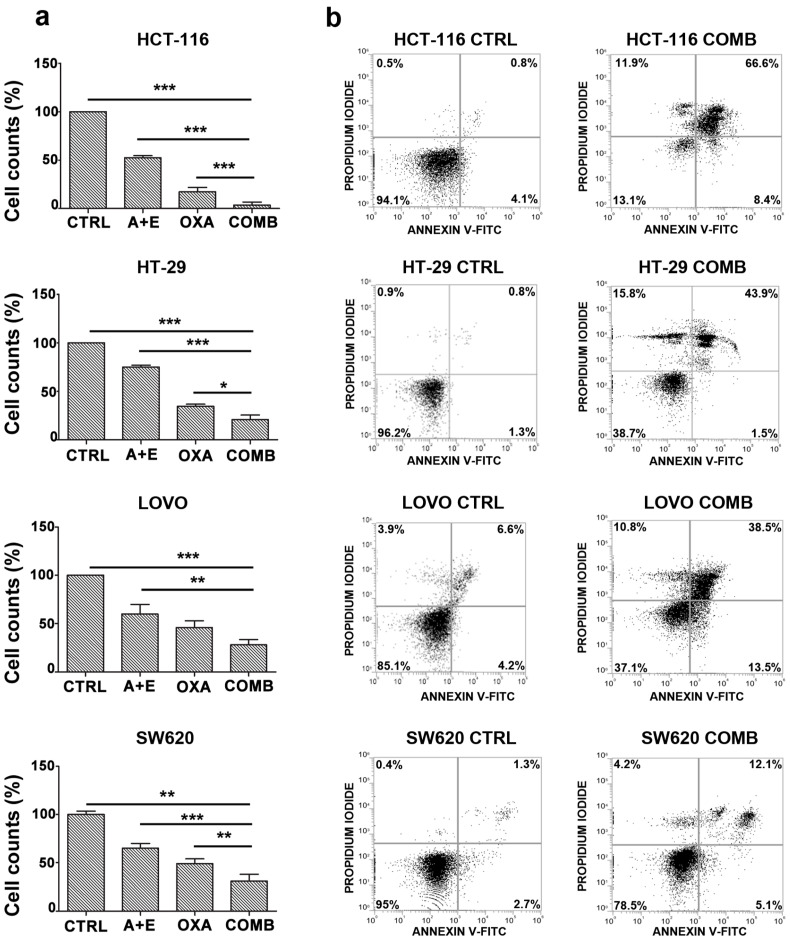
AHCC and ETAS combination enhances oxaliplatinum effects in colon cancer cells (**a**) Flow cytometric absolute counts (with Propidium Iodide) of viable colon cancer cells treated with AHCC and ETAS in combination (AHCC + ETAS), oxliplatinum alone (OXA), or with a combination of AHCC, ETAS and oxaliplatinum (COMB) for 48 h. For HCT-116, HT-29 and SW620 cells, we employed 3 mg/mL AHCC + 0.5 mg/mL ETAS. For LOVO cells we employed 7 mg/mL AHCC + 1.16 mg/mL ETAS. Oxaliplatinum was used at 10 μM for all cell lines. “CTRL” indicates untreated cells. The mean ± SD of three independent experiments is plotted. Asterisks indicate statistically significant differences with respect to different samples. * *p* < 0.05, ** *p* < 0.01, *** *p* < 0.001; (**b**) Flow cytometric analysis of Annexin V-FITC/PI stained CRC cells treated with a combination of AHCC, ETAS and oxaliplatinum (COMB) for 48 h. The percentages of early apoptotic cells (Annexin-V FITC+/PI−; bottom right quadrant) and late apoptotic/necrotic cells (Annexin-V FITC+/PI+; top right quadrant) are shown. “CTRL” indicates untreated cells.

To assess the effectiveness of the combination of AHCC and ETAS treatment as a potential adjuvant strategy to conventional chemotherapy in CRC, we treated colon cancer cells for 48 h with combined AHCC and ETAS, oxaliplatinum as a single agent, or with a combination of AHCC, ETAS and oxaliplatinum. We found a significant decrease of the number of viable cells in all CRC cell lines tested (Figure 6a). In particular, the combination of AHCC, ETAS and oxaliplatinum was significantly more effective in reducing cancer cell growth (Figure 6a) in all cell lines analyzed. To clarify if these effects were due to an increase of apoptotic cells, we evaluated the effects of the combined treatment in CRC cell lines, performing flow cytometric analysis on HCT-116, HT-29, LOVO and SW620 cell lines. In response to treatment with 10 μM of oxaliplatinum and AHCC and ETAS (3 mg/mL AHCC + 0.5 mg/mL ETAS for all cell lines, except for LOVO cells in which we used 7 mg/mL AHCC + 1.16 mg/mL), we detected a marked increase in the percentage of early apoptotic (positive for Annexin V) and/or late apoptotic (positive for both Annexin V and PI) cells after 48 h of treatment in all cell lines (Figure 6b). HCT-116 cells were the most sensitive to the combined treatment with about 70% of apoptotic cells, while SW620 cells were the least sensitive, with 20% of apoptotic cells after the treatment (Figure 6b).

## 3. Discussion

Colon cancer is the second leading cancer worldwide [38]. Although CRC management and therapy are based on surgery, adjuvant radio- and chemotherapy, CRC remains one of the most severe malignancies, especially when it reaches advanced stages [2,46]. Approximately 50% of patients with CRC eventually metastasize; nevertheless, patients with advanced CRC and distant metastasis are not always suitable for conventional intervention and exhibit a poor 5-years survival rate of <10% [3].

Multi-drug conventional therapies have been developed for this disease, leading to significantly improved patient response and overall survival in CRC. However, drug-resistance is very common in the advanced stages of colon cancer [47]. Therefore, novel agents, including natural products, are currently being considered for more efficient cancer treatments and to reduce chemotherapy toxicity in CRC patients.

The use of natural compounds could reduce the adverse effects of anticancer therapy and cancer symptoms, improving patient quality of life. These therapeutic adjuvants have received increasing interest due to the potential to reduce side-effects.

AHCC has been broadly studied for safety in patients with tumors [48,49,50]. Numerous studies have explored the relieving effects of AHCC for chemotherapy-related side-effects. For example, AHCC was able to reduce hematological toxicity of gemcitabine in non-tumor-bearing mice and reduced 6-mercaptopurin and methotrexate-induced liver injury in animal models [18]. Additionally, AHCC enhanced the chemotherapeutic effects of UFT (5-Fluoro-1-(tetrahydrofuran-2-yl)pyrimidine-2,4(1H,3H)-dione) in a model of mammary adenocarcinoma in rats [51], and cisplatin in tumor bearing mice [16]. ETAS is a natural compound that is being evaluated for its beneficial effects in cancer therapies and neurological diseases through the modulation of HSPs [23,25]. Moreover, ETAS showed anti-inflammatory activity induced by reactive species of oxygen [26,27].

Here, we firstly evaluated in vitro efficacy of the administration of AHCC and ETAS in combination in CRC cells. Then, we tested if this combination was able to enhance the effects of a conventional chemotherapeutic drug used in CRC, oxaliplatinum, and was useful as adjuvant supplement for integrative medicine. 

The combination of AHCC and ETAS was synergistic in CRC cell lines, inducing a robust decrease in cell viability in all cell lines employed. On the contrary, the treatment had no significant effects on colonocytes from healthy donors, highlighting that AHCC and ETAS combination is a potential candidate for clinical benefits on cancer cells. We employed biopsies from healthy donors because normal colon cell lines origin from fetal colon and are immortalized cells, more similar to tumor cells. 

Based on the evidence provided by other studies on the role of natural compounds on cancer cells [52], we demonstrated that antiproliferative effects were the result of apoptosis induction. In fact, the cytotoxic effects mediated by the combination of AHCC and ETAS correlated with an induction of apoptosis, as demonstrated by the cleavage of caspases 9, and 3, and PARP, and the modulation of BCL-2, Cytochrome C and p(Ser46)p53. The combination of AHCC and ETAS also decreased HSP27 phosphorylation and activation, which was demonstrated to be correlated to chemotherapy resistance in pancreatic cancer, and associated to poor prognosis in CRC cancer [23,35,36].

Being that the prognosis of CRC metastatic patients is very poor [38], the discovery of novel compounds, which could reduce cellular migration, is an urgent need in CRC. Interestingly, we found a significant reduction of cell motility and migration after treatment with AHCC and ETAS, accompanied by a downregulation of ROCK2 protein expression levels, one of the crucial players in cancer migration. In fact, ROCK2, a serine-threonine kinase, is involved in cytoskeleton organization, regulation of actomyosin contractility, formation of stress fibers, and turnover of focal adhesions [53]. ROCK2 is overexpressed and associated with increased invasion and poor survival in several tumors, including colon cancer [39,40,41]. Conversely, ROCK2 inhibition suppresses invasion and metastasis formation [54,55].

We also found a significant decrease in MMP9 protein levels, and this is of potential interest, due to the fact that the overexpression of MMP9 and other metalloproteases are associated to a worse outcome in CRC cancer, and found MMP9 significantly more expressed in CRC patients compared to healthy donors [42,43]. AHCC and ETAS combined treatment also induced a significant increase of E-cadherin protein expression, whose downregulation has been previously associated with invasiveness and migration abilities in colon cancer stem cells [45]. All these obtained data pointed out the potential role of these compounds in decreasing metastatic potential of CRC cells.

It has been extensively demonstrated that among CRC cells it is possible to identify CSCs, a cell population with replicative immortal and self-renewing properties [56]. In addition to the role in tumor growth and homeostasis, CSCs are thought to be fundamental in metastatic processes. The essential role of CSCs in the metastasizing process is demonstrated by findings which show that metastasizing cells acquire stem cell properties, for example, through epithelial-to-mesenchymal transition (EMT) [57,58,59]. Consequently, selective depletion of CSCs in primary tumors has been demonstrated to protect from the appearance of distant metastases [60]. For these reasons, CSCs represent a critical target for pharmaceutical intervention. In this context, we tested the effectiveness of AHCC and ETAS in combination on HT-29 and SW620-derived CSCs spheres. The preliminary data showed a potential role of the combination of AHCC and ETAS in inducing a decrease in CSCs viability, but the concentration employed for 2D cultures was not sufficient to gain a significant effect on proliferation in tumorspheres, which mimic the physio-pathological condition in the tumor. 

On the other hand, the AHCC/ETAS combination was able to induce a significant downregulation of *LGR5* gene expression in CSCs from SW620. High expression of *LGR5*, a specific marker for the stemness, is associated with a metastatic phenotype and poor prognosis in CRC [61]. The downregulation of *LGR5* obtained after the combined treatment, suggests a potential induction of cell differentiation. Indeed, *LGR5* expression is lost during the physiological intestinal differentiation [62]. Moreover, a recent study demonstrated that *LGR5* is involved in the maintenance of distant metastases in CRC [63].

We also detected a significant decrease in *Notch1* gene expression in CSCs from the SW620 cell line. *Notch1* has been demonstrated to be dysregulated in CRC, correlating with poor survival, CSC phenotype and EMT, thus resulting in tumor progression [64]. The reduction of *Notch1* gene expression that we observed in CSCs reinforces the hypothesis that it can be considered a possible target for anticancer therapies. 

Finally, we demonstrated that the combination of AHCC and ETAS was effective in enhancing the effects of oxaliplatinum, a chemotherapeutic drug, typically employed in CRC therapy. 

The combined treatment of AHCC and ETAS induced a significant increase in apoptosis, sparing colonocytes from healthy donors, and was able to induce a strong reduction in migration potential, accompanied by a significant modulation of proteins involved in invasiveness. The combined treatment was able to significantly enhance the cytotoxic effects of oxaliplatinum, paving the way to new possible combinations of integrative medicine, administered with conventional chemotherapeutical drugs. 

## 4. Materials and Methods

### 4.1. AHCC and ETAS Preparation

AHCC^®^ is a standardized extract of cultured *Lentinula edodes* mycelia, while ETAS^®^ is a standardized extract of *Asparagus officinalis* stem. AHCC and ETAS were provided by Amino Up Co., Ltd. (Sapporo, Japan). Compounds were freshly prepared by dissolving into DMEM media at a final concentration of 25 mg/mL for AHCC (as AHCC-FD powder) and 20 mg/mL for ETAS (as ETAS50 powder). After dissolving, the solution was passed through a 0.22-micron filter (Sarstedt AG & Co. KG, Numbrecht, Germany) and used at different concentrations for AHCC and ETAS.

### 4.2. Cell Line Cultures and Treatments

Cell lines were obtained from DSMZ or ATCC and were routinely tested for mycoplasma contamination using the Mycoalert detection kit (Lonza, Basel, Switzerland). Cell lines were cultured in DMEM high glucose (HCT-116 and HT-29), RPMI (LOVO), or Leibowitz’s L 15 (SW620 cells) medium (Thermo Fisher Scientific Inc., Rockford, IL, USA) supplemented with 10–20% of Fetal Bovine Serum (FBS) (Thermo Fisher Scientific Inc., Rockford, IL, USA), 100 U/mL penicillin, and 100 μg/mL streptomycin (Sigma-Aldrich, Saint Louis, MO, USA) at 37 °C, in a humidified atmosphere of 5% CO_2_. SW620 cells were cultured in Leibowitz’s L-15 medium, formulated for use in CO_2_-free systems. Cells were treated with a combination of AHCC and ETAS at the following concentration: AHCC 3 mg/mL + ETAS 0,5 mg/mL or AHCC 7 mg/mL + ETAS 1.16 mg/mL for 48 h. Oxaliplatinum (from Sigma-Aldrich, Saint Louis, MO, USA) was employed at 10 μM.

### 4.3. Human Tissue Collection and Primary Cell Culture 

Biopsies of colorectal normal mucosa were obtained from 11 healthy patients during routine endoscopy at the University Hospital of Modena. The study was approved by the competent Ethic Committee and the Local Health Agency of Modena, Italy. Every patient enrolled in the study signed a detailed written informed consent. The study was carried out according to the Declaration of Helsinki, to the Good Clinical Practice principles for medical research and to the current regulations relating to the protection and processing of personal and sensitive data (European Regulation n. 679/2016). Three samples of normal colorectal mucosae were collected for each patient using standard endoscopic biopsy forceps (Endo Jaw 2.8 mm, Olympus, Hamburg, Germany) and immediately placed in 2 mL of Intesticult Organoid Growth Medium (Human) (Stemcell Technologies, Vancouver, CA, USA). 

To isolate crypt/gland units, surgical specimens were minced with a scalpel and the cell mixture was pipetted up and down several times to dissociate the cells. Cells were seeded (7 × 10^4^ cells per well) in a 96-well plate and incubated with Organoid Growth Medium and cultured for 72 h in a humidified atmosphere of 5% CO_2_.

### 4.4. Cell Viability Analysis in Cell Lines

Cell proliferation following treatment with single agents or combined treatment was assessed using the MTT [3-(4,5-Dimethylthythiazol-2-yl)-2,5-diphenyltetrazolium bromide] cell proliferation kit (Roche Diagnostic, Basel, Switzerland), according to the manufacturer’s instructions. The combination effect and a potential synergy were evaluated from quantitative analysis of the dose-effect relationships, as described by Chou and Talalay, using CompuSyn software [65]. Concentration- and time-dependent effects on cell viability of AHCC and ETAS (as a single agent or in combination) were determined through flow cytometry absolute count of viable cells, employing the 123count eBeads and propidium iodide staining (Sigma Aldrich, Saint Louis, MO, USA) to identify cell death. 

### 4.5. Treatments of Primary Cells from Biopsies

The cells obtained from patient biopsies were treated with a combination of AHCC and ETAS at the following concentration: AHCC 3 mg/mL + ETAS 0.5 mg/mL for 48 h. For each patient, the treatment was performed in triplicate. The effects on cell viability of the AHCC and ETAS combination were determined through the RealTime-Glo™ MT Cell Viability Assay (Promega, Fitchburg, MA, USA), according to standard procedures.

### 4.6. Isolation of Cancer Stem Cells from Tumor Cell Lines

Once semi-confluence was reached in the standard medium, cells belonging to HT-29 and SW620 tumor cell lines were detached from the flasks by trypsin-EDTA and seeded at a density of 10^4^ cells/mL in serum-free selective medium composed by DMEM/F12 (Sigma-Aldrich, Inc., St. Louis, MO, USA) added with 1 mg/mL EGF (Sigma-Aldrich, Inc., St. Louis, MO, USA), 10 µg/mL FGF (Gibco, Carlsbad, CA, USA) and 1% vitamin B27 (Gibco, Carlsbad, CA, USA) in order to induce selection of cancer stem cells (CSCs), able to grow in 3D tumorsphere suspension cultures. After three weeks, the supernatant with CSCs spheres was collected, centrifuged 10 min at 800 rpm, then treated by trypsin-EDTA and by pipetting for cell enzymatic and mechanical disaggregation. The selection of CD326^+^ cells, presumably CSCs, was carried out using the S-pluriBead kit (pluriSelect GmbH, Leipzig, Germany), which allows the selective cell sorting by filtering cells that are bound to beads conjugated to a specific antibody. Isolated cells, once detached from beads, were immediately re-cultured 1:3 in order to obtain a selective and considerable expansion of CSC spheres.

### 4.7. Annexin V-FITC/PI Staining 

Cells were treated with AHCC and ETAS (alone or in combination) for 48 h and apoptosis analyses were performed through the Annexin V-FITC Apoptosis Detection kit (eBioscience, Thermo Fisher Scientific, Waltham, MA, USA), according to the manufacturer’s instructions. Analyses were performed on an FC500 flow cytometer (Beckman Coulter, Brea, CA, USA) with the appropriate software (CXP, Beckman Coulter vers 2.2). At least 10,000 events per sample were acquired.

### 4.8. Immunofluorescence Microscopy 

From each patient, 10^4^ cells were obtained and were seeded on two-well chamber glass slides for immunofluorescence analysis. Cells were treated with AHCC and ETAS for 48 h. At the end of the incubation period, cells were fixed in 4% paraformaldehyde and incubated with anti-human Cytokeratin-18 (EXBIO, Vestec, Czech Republic) primary antibody overnight at 4 °C. Coverslips were next washed with 1× PBS and incubated with fluorescence-labeled secondary antibody Alexa Fluor-594 (Thermo Fisher Scientific Inc., Rockford, IL, USA) for 1 h at room temperature. Slides were washed and mounted with DABCO 10% solution and were observed by a Nikon A1 confocal laser scanning microscope. The confocal serial sections were processed with ImageJ software to obtain three-dimensional projections, and image rendering was performed using Adobe Photoshop CS 8.0 software (Adobe Systems, San Jose, CA, USA). All the images shown in this paper are representative of at least three independent experiments carried out under the same conditions. To quantify Cytokeratin-18 positive cells, slides were examined at 20× magnification for each condition to count almost 150 cells. Starting randomly, every 3rd field of vision on one slide was used for sampling all cells within the unbiased sampling frame. This absolute sample size is near-optimal independent of the dimensions of the object. A code number was assigned to each condition and the score was determined by an observer who was blind to sample types during analysis.

### 4.9. Western Blotting Analyses

Immunoblotting was performed using standard procedures as previously described [66]. 30 μg of HCT-116 and LOVO cell lysates were separated by SDS– PAGE using 4–20% Criterion TGX polyacrylamide gels (Bio-Rad, Hercules, CA, USA) and blotted onto a nitrocellulose membrane (Bio-Rad). All primary and secondary antibodies were from Cell Signaling Technology, Danvers, MA, USA. Proteins were detected using the ECL, the ChemiDoc-It2 Imaging System and the VisionWorksLS Software for the analysis (UVP, LLC, Upland, CA, USA). Treatments with AHCC and ETAS were performed for 48 h. Representative images of Western blotting were shown.

### 4.10. Wound Healing Assay

Cells (HCT-116, HT-29, LOVO, and SW620) were seeded in 24-well plates and the wound healing assay was performed in non-treated (control) and treated (AHCC and ETAS) cells. A reproducible longitudinal scratch in the monolayer was made the following day using sterile micropipette tips. The process of wound closure was monitored at 0 and 24 h by photographing the central field of the scratches under an inverted light microscope (Olympus CKX41, Olympus Corp, Tokyo, Japan) mounted with a digital camera (C-7070 Wide Zoom, Olympus) at 10× magnification. The pictured field was standardized each time against a horizontal line drawn on the base of the plate passing through the center of each well. Morphometric analysis of cell migration was performed using a computerized image analysis system (Qwin, 3.0 software, Leica Microsystem Imaging Solution, Ltd., Wetzlar, Germany). A region that included the artificial scratch and the adjacent cell monolayer was selected as the standard region of interest (ROI). The wound healing effect was calculated as (1 − Ax/A0)%, where A0 and Ax represented the empty scratch area at 0 and 24 h, respectively.

### 4.11. Transwell Migration Assay

Cell migration assay kits (CytoSelect assays) were purchased from Cell Biolabs (San Diego, CA, USA). We employed 8 μm pore size polycarbonate membrane inserts to evaluate cell migration abilities. Briefly, 500 μL of culture medium containing 20% FBS was added to the lower chambers. Cells were resuspended in 300 μL of culture medium with 1% FBS and a combination of AHCC and ETAS (AHCC: 7 mg/mL and ETAS: 1.16 mg/mL) was seeded on the top of the membrane of each well. HCT-116 were seeded at a final density of 75,000 cells, while HT-29, SW620 and LOVO were seeded at a final density of 300,000 cells. Cells were incubated for 24 h, at 37 °C in 5% CO_2_ in a humidified atmosphere. At this time point, cells were incubated with Cell Stain Solution for 15 min at room temperature. Then, Extraction Solution was added and incubated for 15 min to allow cell lysis. OD at 570 nm was measured in an ELISA plate reader (Bio-Rad, Hercules, CA, USA). Data were plotted as percentage of drug-treated migrated cells versus control migrated cells.

### 4.12. Proteome Profiler

HCT-116 and LOVO cells were treated with a combination of AHCC and ETAS for 48 h. At this time point, human cell stress proteins were detected using the Proteome Profiler human cell stress array kit (R & D systems, Minneapolis, MN, USA) according to the manufacturer’s instructions. Proteins were captured using the ECL, the ChemiDoc-It2 Imaging System and the VisionWorksLS Software for the analysis (UVP, LLC, Upland, CA, USA).

### 4.13. CSCs Treatment

CSCs derived from each cell line (HT-29 and SW620) were seeded in a 6-well microplate at a density of 10^4^ cells per well and treated with the following solution: 7 mg/mL AHCC + 1.16 mg/mL ETAS. All treatments were obtained dissolving the stock solution of AHCC (25 mg/mL) and ETAS (20 mg/mL) in serum-free medium, selective for spheres, composed by DMEM/F12 (Sigma-Aldrich, Inc., St. Louis, MO, USA) added with 1 mg/mL EGF (Sigma-Aldrich, Inc., St. Louis, MO, USA), 10 µg/mL FGF (Gibco, Thermo Fisher Scientific Inc., Rockford, IL, USA) and 1% vitamin B27 (Gibco, Thermo Fisher Scientific Inc., Rockford, IL, USA). This medium was used as a negative control. All treatments were performed in triplicate. After 48 h of treatment, the cells were collected and processed for RNA extraction

### 4.14. RNA Extraction, Reverse Transcription, and Quantitative RT-PCR

Total RNA was isolated from cells using the RNeasy Mini Kit (Qiagen, Hilden, Germany) according to the manufacturer’s instructions. Total RNA concentration and quality were measured using a NanoDrop 2000 spectrophotometer (Thermo Scientific, Waltham, MA, USA). cDNA was obtained using the Quantitec Reverse Transcription Kit (Qiagen, Hilden, Germany) starting from 1 µg of total RNA of each sample and was amplified by real-time quantitative polymerase chain reaction (PCR) using the Power SYBR^®^ Green Master Mix™ (Life Technologies, Foster City, CA, USA) and specific forward and reverse pre-designed assays (Sigma-Aldrich, Inc., St. Louis, MO, USA). Primer sequences for cancer stem cell markers are listed in Table 1. 

A preliminary test was carried out to evaluate three potential reference genes (GAPDH, ACTB, TFRC–primer sequence in Table 2). The *GAPDH* gene was selected because its expression was the most stable among treated and untreated cells. The expression profile of a selection of genes was evaluated in order to test the efficacy of treatments (genes and primer sequences in Table 3). 

PCR reactions were performed in 15 µL of final volume containing 7.5 µL of 2× Power SYBR^®^ Green Master Mix™, 100 nM concentration of each primer and 300 nM of cDNA. The amplification reactions were carried out on the ABI PRISM 7500 instrument (Applied Biosystems, Foster City, CA, USA). An initial denaturing step at 95 °C for 10 min was followed by 40 cycles of a two-step profile of 15 s at 95 °C and 60 s at 60 °C. As final step, a melt curve dissociation analysis was performed. A non-template control was used in each assay in order to exclude any biological contaminations.

### 4.15. Statistics

Data are presented as mean values ± SD and were statistically analyzed using a two-tailed unpaired *t*-test or one-way ANOVA and Bonferroni post-test with GraphPad Prism Software (GraphPad, San Diego, CA, USA). A *p* value of <0.05 was considered statistically significant. Gene expression quantification was conducted with the Δ/Δ Ct calculation method [67]. Statistical analysis for the comparison of expression levels between groups of samples was performed using the paired sample *t* test.

## 5. Conclusions

Our study demonstrates that the combination of AHCC and ETAS is efficacious in inducing anticancer effects in CRC cells and suggests the potential use of these compounds as adjuvants for conventional chemotherapy in CRC patients. This data will possibly have an impact on the therapeutic arsenal we have at our disposal against CRC.

## Figures and Tables

**Table 1:** Primer sequences of cancer stem cell markers, used in Real Time (RT)-PCR.

Gene Symbol	Gene Name	Primer Sequences 5′–3′
*OCT4*	POU class 5 homeobox 1	For-CCCGAAAGAGAAAGCGAACC
		Rev-CTCTCGTTGTGCATAGTCGC
*cMyc*	Myc proto-oncogene	For-ATTCTCTGCTCTCCTCGACG
		Rev-TGCGTAGTTGTGCTGATGTG
*CD326*	Epithelial Cell Adhesion Molecule	For-TCTGTGAAAACTACAAGCTGGC
		Rev-GGTTTTGCTCTTCTCCCAAGTT
*CD24*	CD24 molecule	For-CATGGGCAGAGCAATGGTG
		Rev-TGGTGGTGGCATTAGTTGGA
*CD133*	Prominin 1	For-CGACAATGTAACTCAGCGTCTT
		Rev-CACACAGTAAGCCCAGGTAGTA
*NANOG*	Nanog homeobox	For-TGAGTGTGGATCCAGCTTGT
		Rev-TCTCTGCAGAAGTGGGTTGT
*LGR5*	Leucine rich repeat containing G protein-coupled receptor 5	For-AAATGCCTTATGCTTACCAG
		Rev-ATCTTGAGCCTGAAACATTC
*ALDH*	Aldehyde dehydrogenase	For-GACAATGGAGTCAATGAATGG
		Rev-ATCAATTGGTATTGTACGGC
*STAT3*	Signal transducer and activator of transcription 3	For-CCTTTGACATGGAGTTGACC
		Rev-TAAAAGTGCCCAGATTGCTC

**Table 2:** Primer sequences of reference genes.

Gene Symbol	Gene Name	Primer Sequences 5′–3′
*GAPDH*	Glyceraldehyde 3-phosphate dehydrogenase	For-ACAGTTGCCATGTAGACC
		Rev-TTTTTGGTTGAGCACAGG
*ACTB*	Actin beta	For-GGACTTCGAGCAAGAGATGG
		Rev-AGCACTGTGTTGGCGTACAG
*TFRC*	Transferrin Receptor	For-CGCTGGTCAGTTCGTGATTA
		Rev-GCATTCCCGAAATCTGTTGT

**Table 3:** Primer sequences of selected genes, used in Real Time (RT)-PCR.

Gene Symbol	Gene Name	Primer Sequences 5′–3′
*ROCK2*	Rho associated coiled-coil containing protein kinase 2	For-ACTCCATTTTATGCGGATTC
		Rev-CTCCCTATCTGTTAAGAAAGC
*TIMP1*	TIMP metallopeptidase inhibitor 1	For-CACCTTATACCAGCTTATG
		Rev-TTTCCAGCAATGAGAAACTC
*CDH1*	Cadherin 1	For-CTGGGCAGAGTGAATTTTG
		Rev-GACTGTAATCACACCATCTG
*SNAI2*	Snail family transcriptional repressor 2	For-CAGTGATTATTTCCCCGTATC
		Rev-CCCCAAAGATGAGGAGTATC
*ITGA5*	Integrin subunit alpha 5	For-AAGCTTGGATTCTTCAAACG
		Rev-TCCTTTTCAGTAGAATGAGGG
*LGR5*	Leucine rich repeat containing G protein-coupled receptor 5	For-AAATGCCTTATGCTTACCAG
		Rev-ATCTTGAGCCTGAAACATTC
*NOTCH1*	Notch 1	For-AAGATATGCAGAACAACAGG
		Rev-TCCATATGATCCGTGATGTC

## Data Availability

Data is contained within the article and supplementary material.

## Supplementary Materials

The following are available online at https://www.mdpi.com/article/10.3390/ph14121325/s1. Figure S1: AHCC and ETAS treatment does not affect CRC cells at 24 h.
