# Supplementary material for: The Combination of AHCC and ETAS Decreases Migration of Colorectal Cancer Cells, and Reduces the Expression of LGR5 and Notch1 Genes in Cancer Stem Cells: A Novel Potential Approach for Integrative Medicine
Source: Pharmaceuticals (Basel). 2021 Dec 18;14(12):1325. doi: 10.3390/ph14121325 (PMC8705140; doi:10.3390/ph14121325)
Supplement: Supplementary file 1 [file pharmaceuticals-14-01325-s001.zip › pharmaceuticals-1519815-supplementary.pdf]

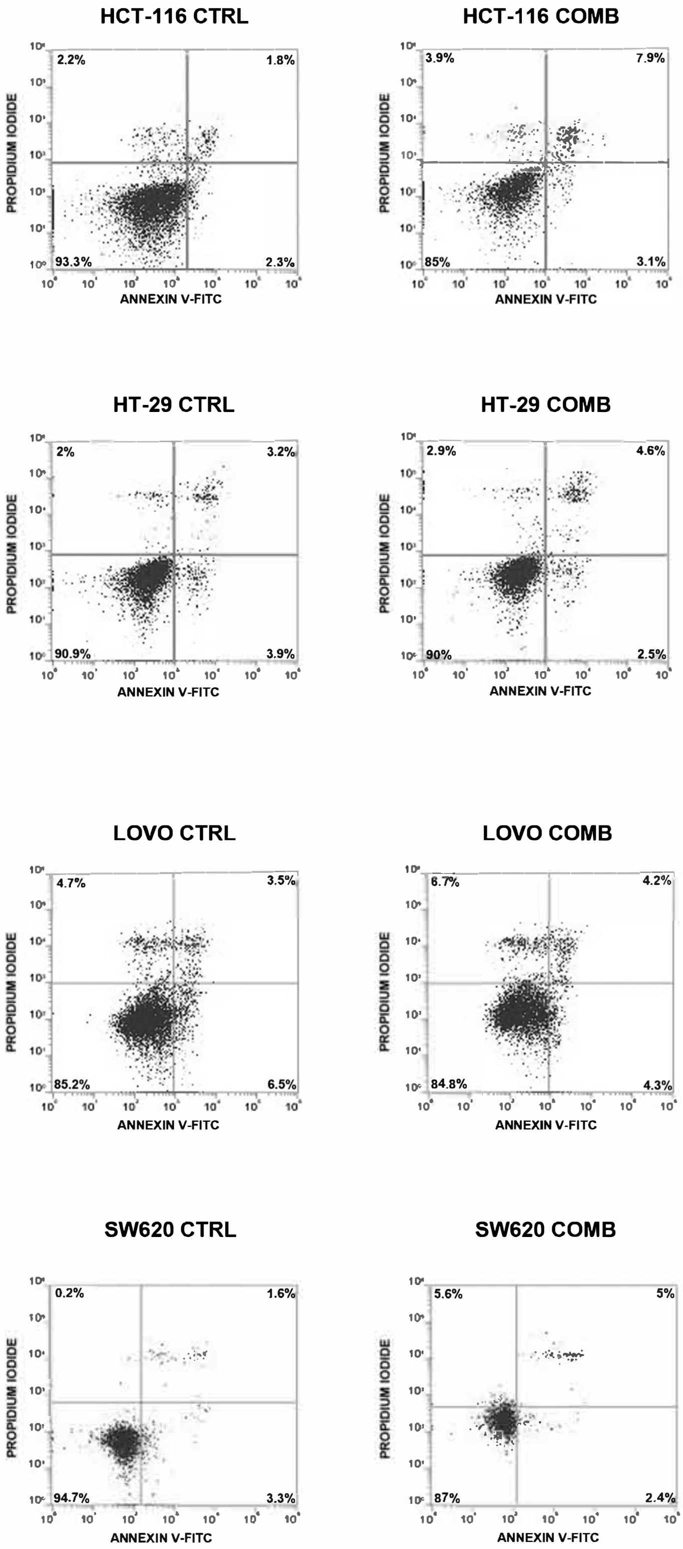

Supplementary Figure S1. AHCC and ETAS treatment does not affect CRC cells at 24 hours (a) Flow cytometric analysis of Annexin V-FITC/PI stained CRC cells treated with a combination of AHCC and ETAS (COMB) for 24 h. The percentages of early apoptotic cells (Annexin-V FITC+/PI−; bottom right quadrant) and late apoptotic/necrotic cells (Annexin-V FITC+/PI+; top right quadrant) are shown. "CTRL" indicates non-treated cells used as control cells.

Supplementary Figure S1
